# Correction: Liu et al. Cholesterol 25-Hydroxylase Suppresses Swine Acute Diarrhea Syndrome Coronavirus Infection by Blocking Spike Protein-Mediated Membrane Fusion. *Viruses* 2023, *15*, 2406

**DOI:** 10.3390/v16060976

**Published:** 2024-06-18

**Authors:** Dakai Liu, Da Shi, Hongyan Shi, Liaoyuan Zhang, Jiyu Zhang, Miaomiao Zeng, Tingshuai Feng, Xiaoman Yang, Xin Zhang, Jianfei Chen, Zhaoyang Jing, Zhaoyang Ji, Jialin Zhang, Li Feng

**Affiliations:** State Key Laboratory for Animal Disease Control and Prevention, Harbin Veterinary Research Institute, Chinese Academy of Agricultural Sciences, Xiangfang District, Haping Road 678, Harbin 150069, China; liudakai0404@163.com (D.L.); shy2005y@163.com (H.S.); zhangliaoyuanzhu@foxmail.com (L.Z.); zhangjiyu0429@163.com (J.Z.); 18790286972@163.com (M.Z.); fengtingshuai@126.com (T.F.); xiaomanyang9766@163.com (X.Y.); zhangxin2410@163.com (X.Z.); chenjianfei@126.com (J.C.); 15204604415@163.com (Z.J.); zy_ji2010@163.com (Z.J.); zhangjialin0106@gmail.com (J.Z.)

In the original publication [1], there was a mistake in Figure 1E as published. These two images were used in a previously published master’s degree article from our laboratory. The corrected Figure 1E appears below. The authors state that the scientific conclusions are unaffected. This correction was approved by the Academic Editor. The original publication has also been updated.

## Figures and Tables

**Figure 1:**
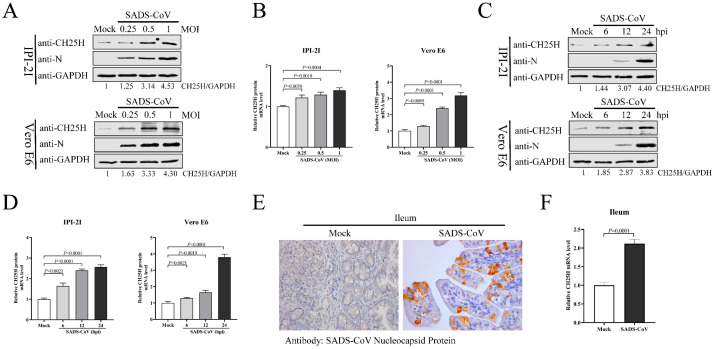
SADS-CoV infection induced CH25H expression in vitro and in vivo. (**A**,**B**) IPI-2I and Vero E6 cells were infected with different doses of SADS-CoV (MOI 0.25, 0.5, or 1). Uninfected cells were used as a control group. Samples from both cell types were harvested at 24 h, and CH25H protein and mRNA levels were determined by Western blotting (**A**) and qRT-PCR (**B**), respectively. (**C**,**D**) IPI-2I and Vero E6 cells were infected with SADS-CoV at MOI 1. Cell samples were collected at 6, 12, and 24 hpi. CH25H protein and mRNA levels were detected by Western blotting (**C**) and qRT-PCR (**D**). (**E**) Representative microphotographs of viral antigen immunochemical staining in SADS-CoV-uninfected and -infected ileal tissues (Bar: 50 μm). (**F**) Total RNA was extracted from ileal tissues, and CH25H mRNA levels were analyzed by qRT-PCR. Means and SD (error bars) of three independent experiments are indicated. *p* values were calculated using two-tailed unpaired Student’s *t*-test.
